# Supplementary material for: Segment Anything for Comprehensive Analysis of Grapevine Cluster Architecture and Berry Properties
Source: Plant Phenomics. 2024 Jun 27;6:0202. doi: 10.34133/plantphenomics.0202 (PMC11208874; doi:10.34133/plantphenomics.0202)
Supplement: Supplementary 1 — Figs. S1 to S6 [file plantphenomics.0202.f1.docx]

**SUPPLEMENTARY FIGURES**


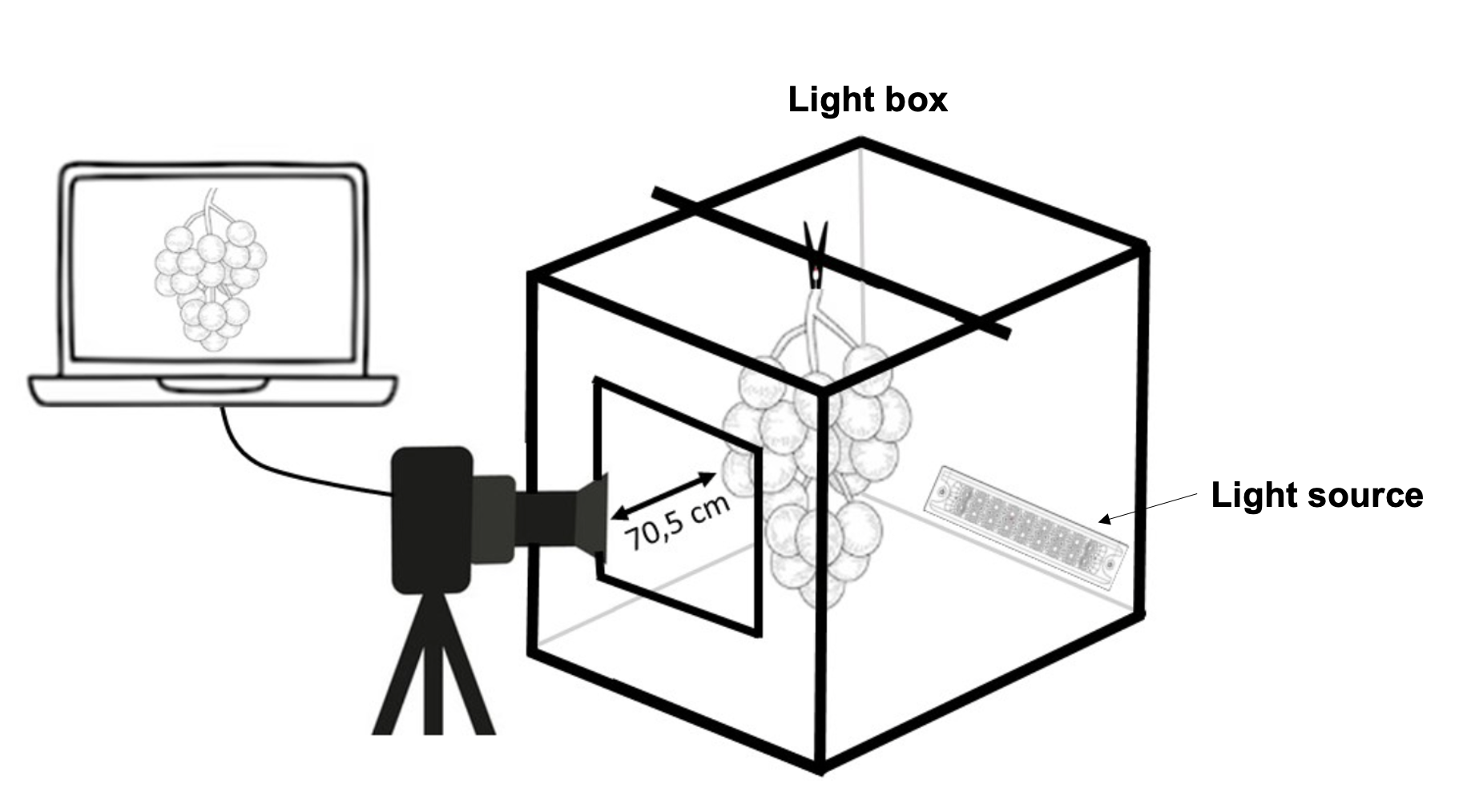


**Fig. S1.** Imaging setup


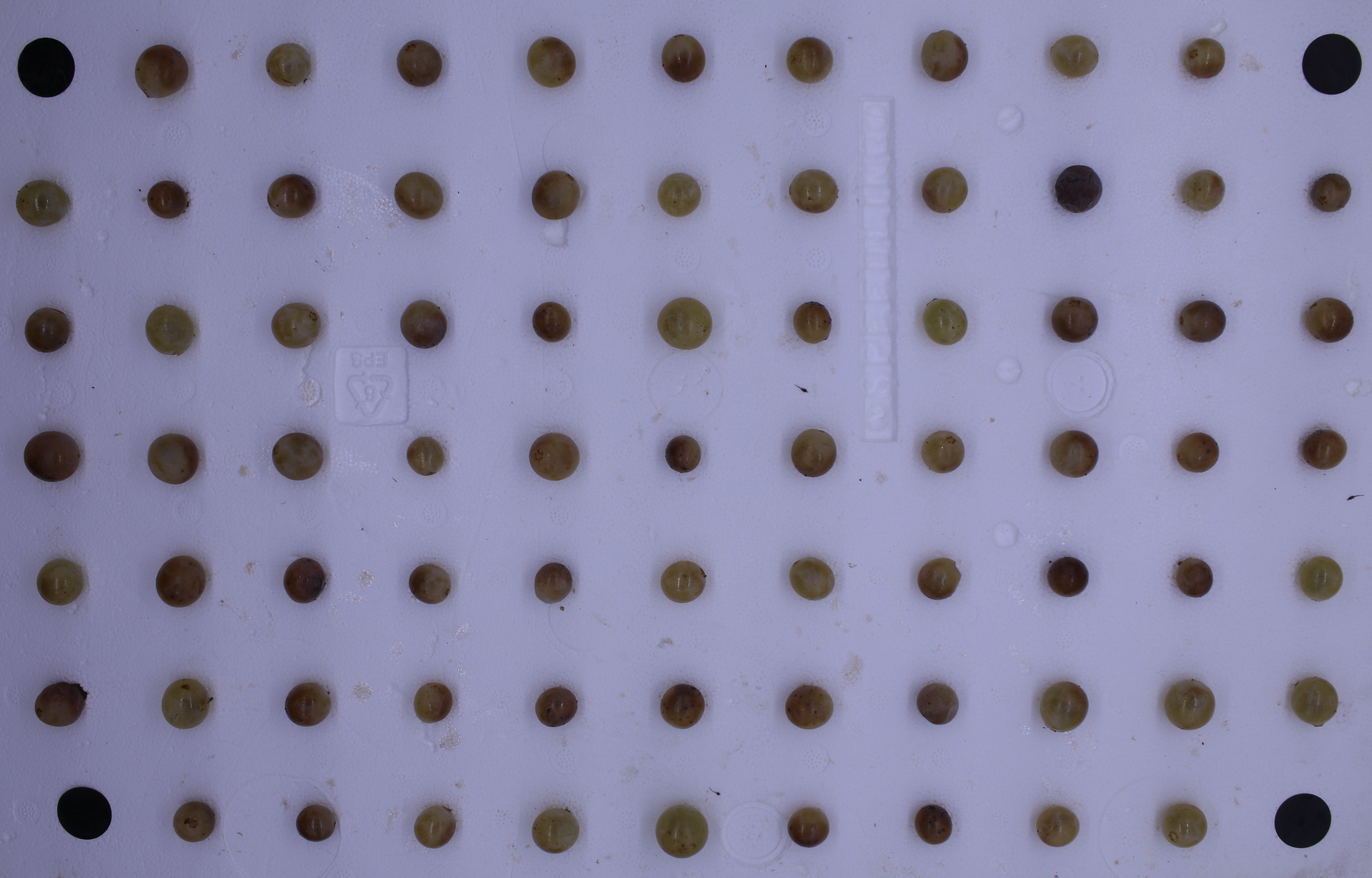


**Fig. S2.** Example of the images with individual berries captured to compare SAM predictions


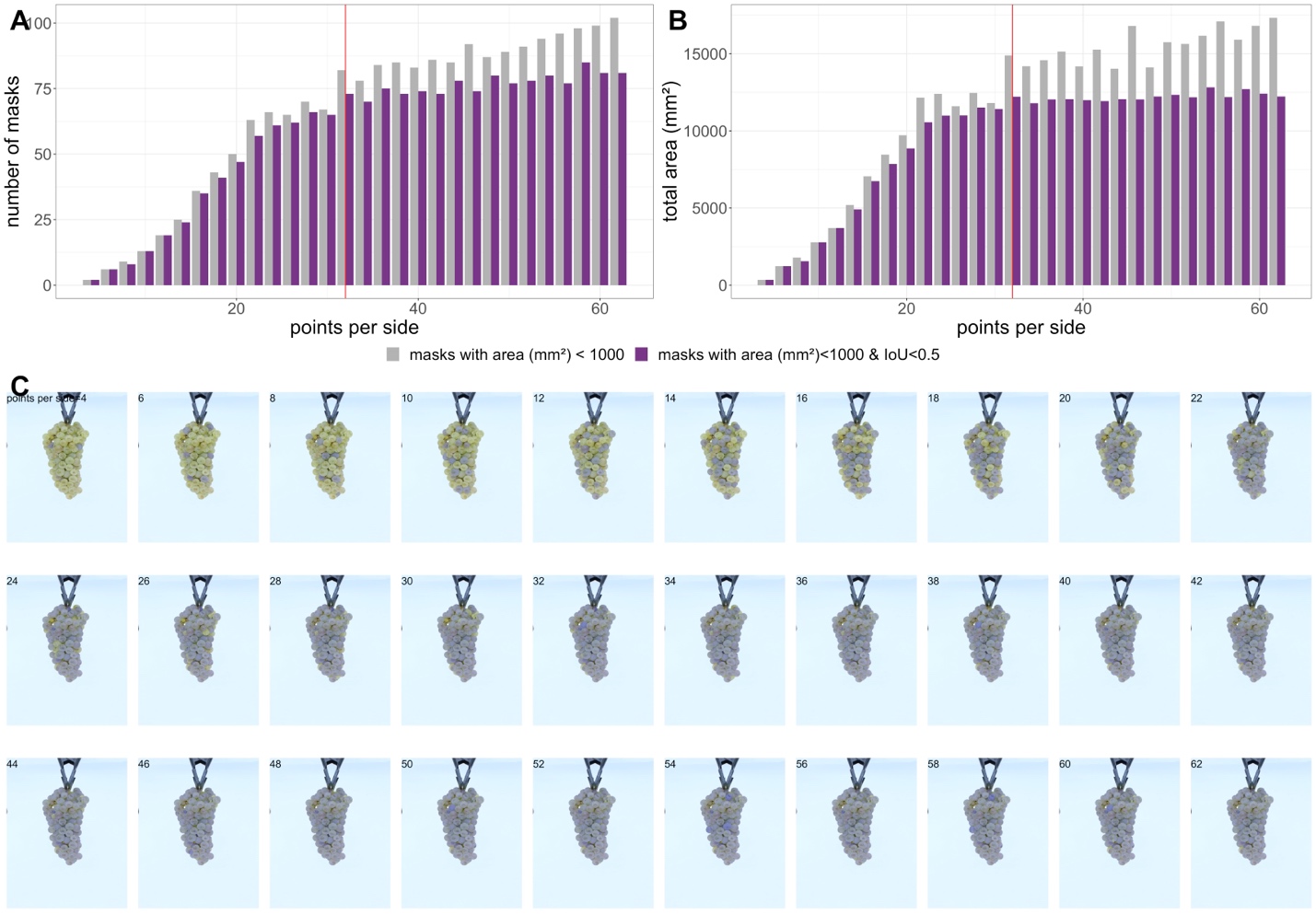


**Fig. S3.** The effect of different point densities in the XY grid used to prompt object detection by SAM. Changes in **(A)** the number of masks and **(B)** the total area depending on the number of points. **(C)** Visual example of an increasing number of objects detected as the grid density increases.


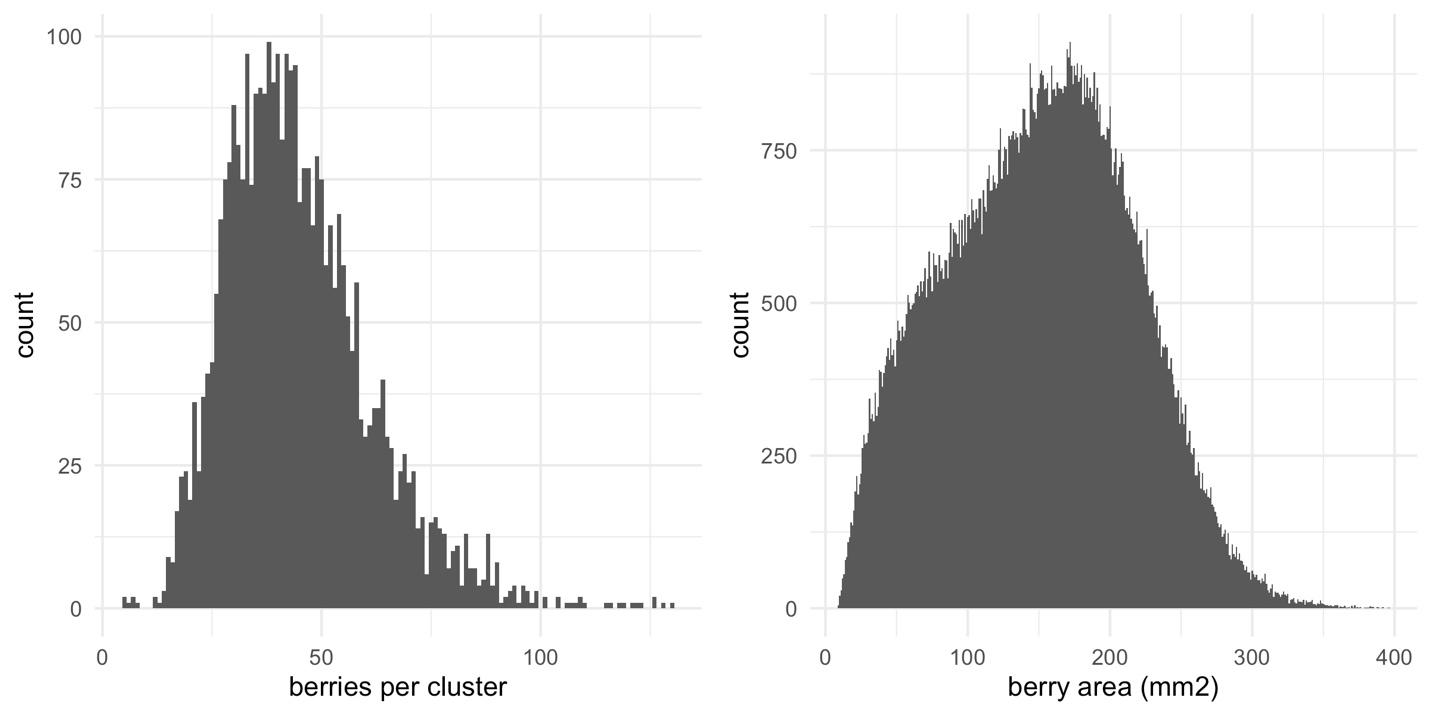


**Fig. S4.** Variation in the number of berries per cluster and berry area.


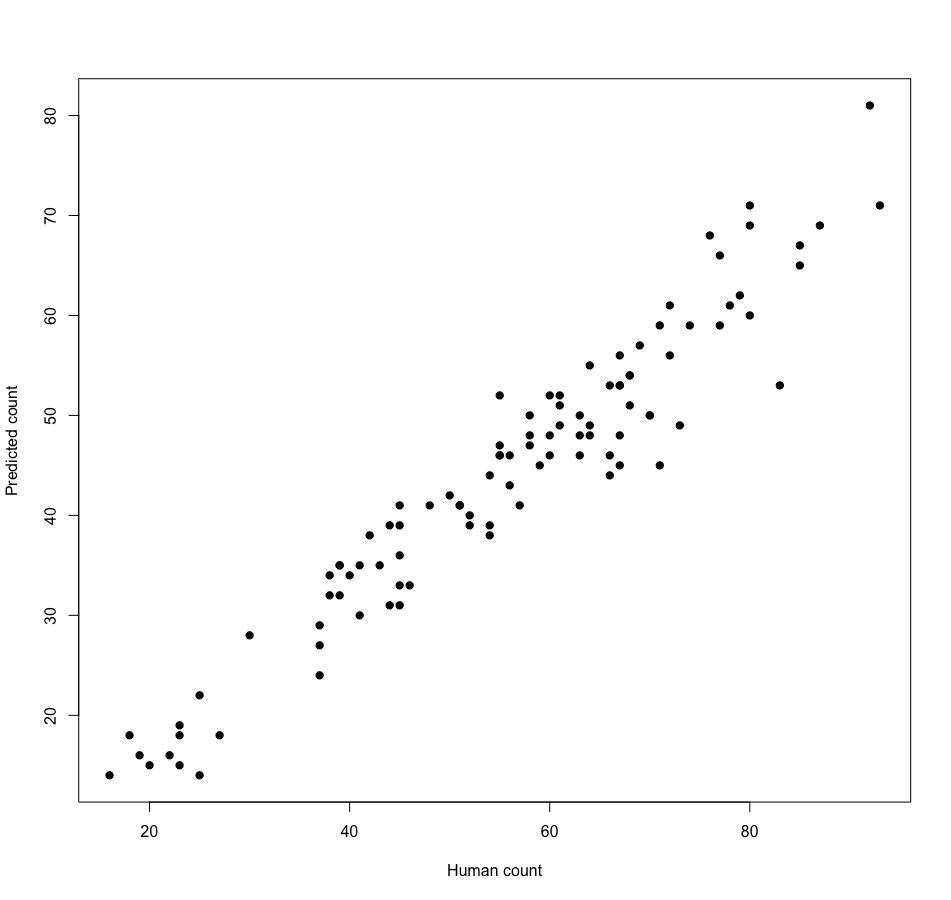


**Fig. S5.** Correlation between berry number determined by humans, and berry number predicted by SAM. Pearson’s correlation coefficient = 0.9619


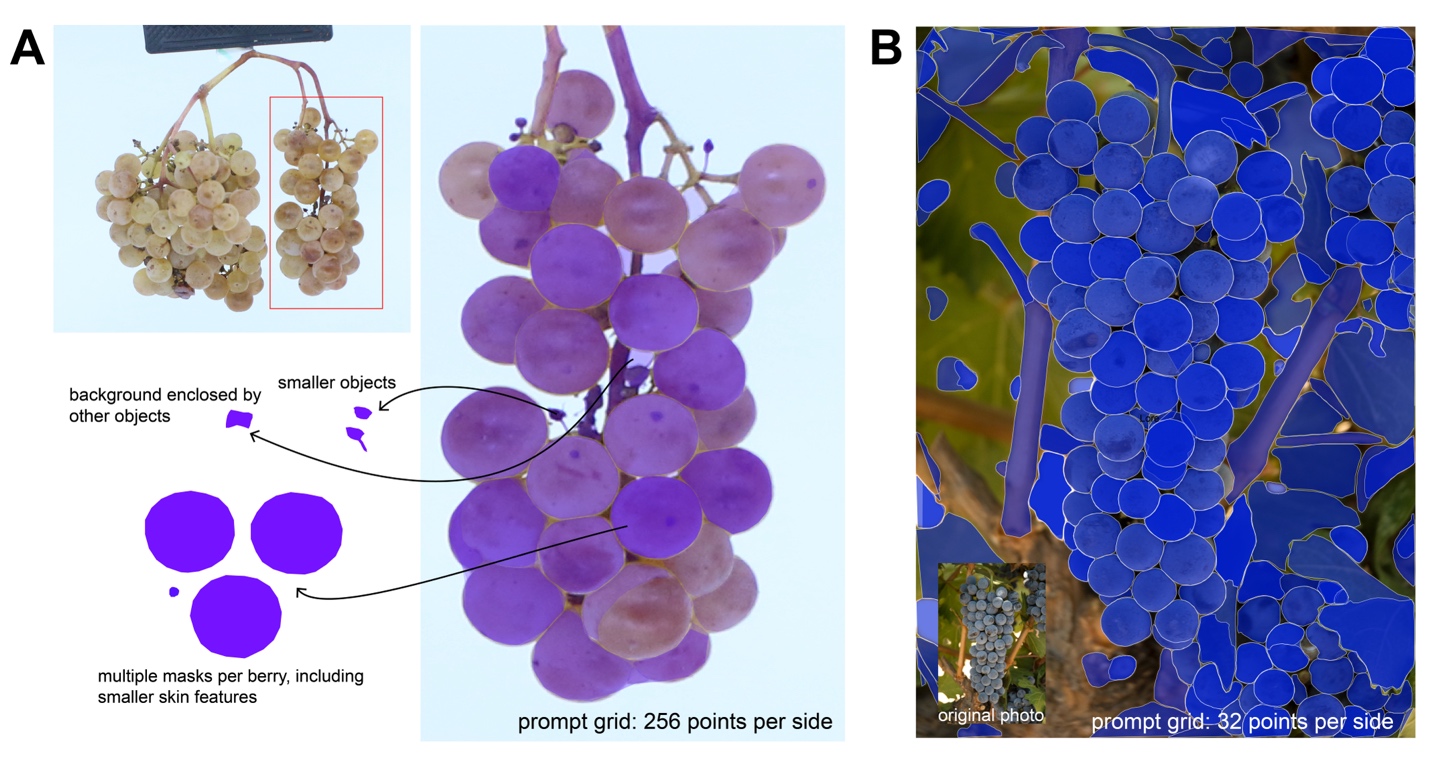


**Fig. S6. Examples of instances where SegmentAnything produces spurious information.** **(A)** Using an extremely high number of points per side (256) in the XY grid used to initialize object search results in smaller features (e.g., skin marks, smaller undeveloped berries, etc.) being detected. IoU might be used to clear out some of the overlapping objects, but it has to be executed in conjunction with other filters such as area (for reducing smaller objects). **(B)** Application of SegmentAnything in a vineyard photograph without prior removal of the background results in many non-berry objects being identified. Raw image taken from <https://fps.ucdavis.edu> with permission.
